# Preparation of tobacco pyrolysis liquids in subcritical/supercritical ethanol and their application in the aroma enhancement of heated cigarettes

**DOI:** 10.3389/fchem.2023.1347215

**Published:** 2024-01-11

**Authors:** Xuebin Zhao, Shengchen Zhao, Yongming Xu, Heng Xu, Zhan Zhang, Haiying Tian, Qiang He, Shengtao Ma, Beibei Gao, Chengjie Ma

**Affiliations:** ^1^ Technology Center, China Tobacco Henan Industrial Co., Ltd., Zhengzhou, China; ^2^ Green Catalysis Center, College of Chemistry, Zhengzhou University, Zhengzhou, China; ^3^ Zhengzhou Tobacco Research Institute of CNTC, Zhengzhou, China

**Keywords:** tobacco, pyrolysis, subcritical/supercritical ethanol, heated cigarettes, aroma-enhancing

## Abstract

For the aroma enhancement research of heated cigarettes, it is worth exploring whether tobacco can be pyrolyzed into pyrolysis liquids containing a large number of volatile aroma components. In this study, tobacco pyrolysis liquids were prepared in subcritical/supercritical ethanol, and their applications in the aroma enhancement of heated cigarettes were investigated. The optimal conditions of supercritical liquefaction reactions were determined by optimizing the reaction time, liquid/solid mass ratio and temperature conditions. Moreover, the effect of supercritical liquefaction conditions on volatile aroma components in tobacco pyrolysis liquids was investigated by GC-MS. The results indicated that the reaction temperature had the most significant impact on the tobacco pyrolysis reaction, and higher reaction temperature promoted the pyrolysis conversion of tobacco, resulting in enhanced tobacco conversion and a high content of volatile components in the tobacco pyrolysis liquid. The optimal reaction conditions for the preparation of tobacco pyrolysis liquid were found to be a temperature of 220°C, a liquid/solid mass ratio = 15, and a 2-h reaction time. Meanwhile, the content of ester compounds and nicotine in the tobacco pyrolysis liquid increased significantly with the increase of reaction temperature. Sub/supercritical ethanol treatment significantly destroyed the surface structure of tobacco, and the degree of tobacco depolymerization increased when temperature rised. The analysis of aroma compounds in the smoke of heated cigarettes indicated that the tobacco pyrolysis liquid could significantly increase the release of aromatic substances and has a significant aroma-enhancing effect. This article proposed and prepared tobacco pyrolysis liquid in subcritical/supercritical ethanol and explored its potential application in the aroma enhancement of heated cigarettes, offering a new route for flavor enhancement technology for this type of product.

## 1 Introduction

Unlike traditional cigarettes, which mainly release aromatic substances through high-temperature combustion, heated cigarettes are a new type of cigarette that use closed heating to distill and pyrolyze tobacco components to release aerosols without combustion (Gasparyan et al., 2018; Li et al., 2023). Their heating temperature is generally less than 350 ^o^C and only distillation and simple pyrolysis reactions occur, which effectively mitigate the generation of toxic and potentially toxic compounds that are harmful to humans and the environment (Caputi, 2017; Farsalinos et al., 2018; El-Kaassamani et al., 2022; Ardati et al., 2023). However, due to the low applied temperature and the scarcity of the tobacco pyrolysis reaction, the quantity and concentration of released smoke and fragrance components are low, especially the original aroma of tobacco, resulting in an extremely different sensory experience from traditional cigarettes (Bekki et al., 2017). Therefore, the development of flavoring technology is an urgent problem to be solved in the research process applied to heated cigarettes. The most commonly used technology for cigarette flavoring is the addition of flavors and fragrances such as tobacco extract (Zhang et al., 2014; Harris et al., 2015; Duan et al., 2016; Docheva et al., 2017; Zhao et al., 2022). However, traditional spices often contain some macromolecular aroma substances or precursors, which are difficult to volatilize or pyrolyze in heated cigarettes. Therefore, the effect of using conventional spices directly to enhance the aroma of heated cigarettes is often not ideal (Guan et al., 2022). Considering that aroma components can be produced by tobacco pyrolysis, it is a new route worth exploring to enhance the aroma of heated cigarettes by selecting a reasonable reaction system to promote the pyrolysis reaction of tobacco leaves. In this system, tobacco pyrolysis liquid containing a large number of volatile components could be obtained and added to heated cigarettes.

In recent years, supercritical technology has attracted great attention from researchers due to its unique advantages (Zeng et al., 2014; Li et al., 2015; Malaret et al., 2020; Liu et al., 2021; Yang et al., 2021; Orita et al., 2022). Compared with conventional methods, solvents exhibit many unique properties under supercritical conditions, such as low viscosity, high dispersion, strong solvent solubility, and high thermal conductivity. In the field of biomass catalytic conversion, the pyrolysis and liquefaction of cellulose, lignin, and various biomass resources by utilizing the solubility and generated free radicals of supercritical fluids to obtain high-value-added products has always been a research hotspot (Onwudili and Williams, 2013; Yakaboylu et al., 2015; Casademont et al., 2018; Li et al., 2019; Bartolomei et al., 2021; Leong et al., 2021; Perez et al., 2022). As a special biomass resource, tobacco is mainly composed of aromatic substances and aroma precursors wrapped in cell walls composed of hemicellulose, cellulose, lignin, pectin, etc. (Jiang et al., 2016; Luo et al., 2021; Yang et al., 2022; Yu et al., 2022; Zhang et al., 2022; Yu et al., 2023). The strong solubility of supercritical fluids can not only extract small molecules wrapped in tobacco cells to release aroma, but it can also promote the depolymerization and dissolution of cellulose and lignin to accelerate the pyrolysis and liquefaction of tobacco leaves. Meanwhile, free radicals generated under supercritical conditions can further promote the pyrolysis reaction of aroma precursors in tobacco leaves, producing aromatic substances. Therefore, supercritical fluid would be a reasonable reaction system to promote the pyrolysis reaction of tobacco leaves.

For the aroma-enhancing research of heated cigarettes, it is worth exploring whether tobacco can be pyrolyzed into pyrolysis liquids containing a large number of volatile aroma components. In this study, tobacco pyrolysis liquids were prepared in subcritical/supercritical ethanol, and their application in the aroma enhancement of heated cigarettes was investigated. The conditions of supercritical liquefaction reactions were optimized, and the effect of supercritical liquefaction conditions on volatile aroma components in tobacco pyrolysis liquids was investigated by GC-MS. Furthermore, the aroma-enhancing effect of tobacco pyrolysis liquid was determined by analyzing the aroma substances in heated cigarette smoke. In this article, we prepared tobacco pyrolysis liquid using supercritical fluids and explored their potential application in the aroma enhancement of heated cigarettes for the first time in order to develop a new route for flavor enhancement technology.

## 2 Experiment Section

### 2.1 Materials

Tobacco raw materials (moisture content 10.11%) were provided by China Tobacco Henan Industrial Co., Ltd., which were crushed and screened through a 60 mesh screen; Anhydrous ethanol (chromatographic grade, Tianjin Damao Chemical Reagent Factory). Experimental facilities such as high-pressure reactor (Parr4790, American Parr Company), electronic balance (FA1204B, Shanghai Youke Instrument Co., Ltd.), rotary evaporator (RE-2000A, Zhengzhou Zhengsheng Instrument & Equipment Co., Ltd.) are also used.

### 2.2 Preparation experiments of tobacco pyrolysis liquids in subcritical/supercritical ethanol

Tobacco pyrolysis liquids were prepared by using ethanol as a solvent in a Parr reactor. Typically, 2.00 g of tobacco powder was placed in a 100-mL Parr reactor, a certain amount of ethanol was added, and the reactor was sealed. Pure nitrogen was added and purged three times to completely replace the air in the reactor. Then, the temperature was raised to the target reaction temperature of 3 ^o^C min^−1^ with vigorous stirring. The reactor was quickly cooled by placing it in water after staying for a certain time at the target reaction temperature. Then, the gas in the reactor was released, and the reaction mixture in the reactor was filtered. The solid residue obtained was washed with ethanol, dried, and weighed to calculate the solid residue yield. The liquid sample was evaporated at 40°C and 50 Pa to remove the ethanol solvent (until the sample mass did not change) and weighed. Finally, the liquid sample was diluted with ethanol to obtain tobacco pyrolysis liquid with a 10 wt% content. As a control group, tobacco powders were also refluxed and extracted by ethanol under atmospheric pressure (the reaction temperature was 78°C), and the subsequent processing methods were the same as the above steps to obtain tobacco extract with a 10 wt% content. The calculation formulas for tobacco conversion rate and product yield were as follows:

$$C_{T}=\frac{m_{T}-m_{S}}{m_{T}}\times 100\%$$

$$Y_{L}=\frac{m_{L}}{m_{T}}\times 100\%$$

$$Y_{S}=\frac{m_{S}}{m_{T}}\times 100\%$$

where *Y*_*L*_ is the yield of tobacco pyrolysis liquid, % (mass fraction); *Y*_*S*_ is the yield of solid residue, % (mass fraction); *C*_*T*_ is the conversion rate of tobacco powder, %; *m*_*T*_ is the dry basis weight of tobacco powder raw material, g; *m*_*L*_ is the mass of liquid product after rotary evaporator, g; *m*_*S*_ is the mass of solid residue, g.

### 2.3 Reaction product analysis

The surface morphology of the solid residue was analyzed by a Zeiss field emission scanning electron microscope (Sigma 500). The functional groups in the solid residues were investigated by FT-IR technology using a Nicolet IR 200 infrared spectrometer with potassium bromide tablet treatment, a scanning range of 4,000–400 cm^−1^, and a scanning speed of 64. Elemental analysis was carried out using an Elementar Unicube instrument in CHNS analysis mode. The typical test parameters were as follows: helium and oxygen (with gas purity greater than 99.99%) pressures were set at 1,200 bar, the combustion tube temperature was set at 1150°C, and the reduction tube temperature was set at 850°C. The helium flow rate was 200 mL/min, and the oxygen gas flow rate was 15 mL/min. A thermal conductivity detector (TCD) with a temperature of 65°C was employed. Upon activation, the detector required 45–60 min to stabilize, and testing could commence when the baseline was essentially stable. In the O analysis mode, only 200 mL/min of helium was introduced, while the other conditions remained the same as described above.

The liquid phase products were analyzed by GC-MS equipped with a DB-5MS (60 m × 250 μm × 0.25 µm) capillary column. Generally, 1.00 g of tobacco pyrolysis liquid was diluted with 4.00 g of ethanol containing an internal standard (ethyl salicylate, 100 ppm), followed by thorough shaking and homogenization. A total of 1 mL of solution was taken for analysis, and the parameters were as follows: carrier gas was high-purity He at a flow rate of 1.0 mL/min, with a split ratio of 15:1, and an injection volume of 1.0 μL. Automatic sampling mode was adopted, and the temperature program of the column oven was as follows: the initial temperature was 50°C and held for 1.5 min, then the temperature was raised to 280°C and held for another 25 min at a rate of 8°C/min. The solvent delay was set to 4.8 min for the mass spectrometry detector. The NIST database was used for qualitative analysis of compounds, and the peak area ratios of the compound to the internal standard were used for semi-quantitative analysis.

The quantitative analysis of nicotine in tobacco pyrolysis liquid was calculated by gas chromatography. Typically, 1.00 g of tobacco pyrolysis liquid was diluted with 4.00 g of isopropanol containing an internal standard (heptadecane, ppm), and the test conditions of gas chromatography and the calculation method of nicotine content were referred to in previous reports (Dermody et al., 2015; Wang Ziyan et al., 2020).

### 2.4 Analysis of volatile aroma components in the smoke of heated cigarettes

Volatile aroma component analysis of heated tobacco products was performed by smoking machines and GC-MS/MS methods. A NSM100 heated cigarette smoking machine was used. The smoking interval was 30 s, the smoking duration was 2 s, and the smoking capacity was 55 mL. After heating and smoking the cigarette, a Cambridge filter was folded and placed in a 12 mL sample bottle. 8 mL of dichloromethane was added along with 50 μL of internal standard solution, and the mixture was sonicated for 30 min. The extract was then filtered using a disposable filter (an injection syringe with an organic phase filter membrane), and the filtrate was collected. 1 mL of the cigarette smoke solution was taken in parallel into two chromatography vials, and 50 μL of the analyte protectant was added for GC-MS/MS analysis. The GC-MS/MS analysis of the volatile aroma components were referred to in a previous report (Si et al., 2021).

## 3 Results and discussion

### 3.1 Optimization of preparation conditions for tobacco pyrolysis liquids in subcritical/supercritical ethanol

**FIGURE 1:**
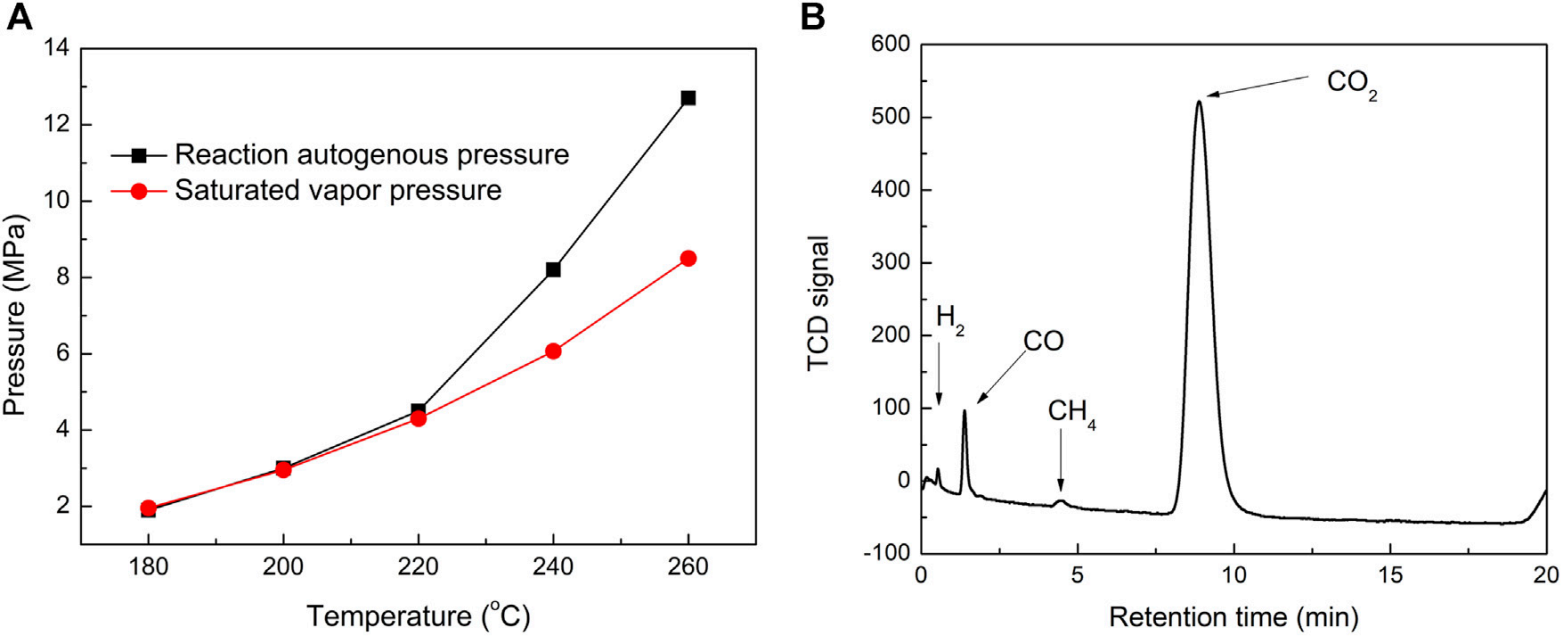
Comparisons of the reaction autogenous pressure and the corresponding saturated vapor pressure of ethanol at different reaction temperatures **(A)**. GC chromatogram of gaseous products collected when the reactor was quickly cooled by being placed in water after reaction at 240^o^C **(B)**.

The influences of experimental conditions such as reaction temperatures, liquid/solid mass ratio, and reaction time on the pyrolysis reaction of tobacco in subcritical/supercritical ethanol were investigated. First, under the condition that the liquid/solid mass ratio was 25 and the reaction time was 2 h, the reaction temperatures of 180°C, 200°C, 220°C, 240°C, and 260°C were employed to investigate the pyrolysis behavior of tobacco, considering that the supercritical temperature and pressure of ethanol were 243 ^o^C and 6.4 MPa, respectively (Kim et al., 2012). The ethanol solvent was in a subcritical state when the reaction temperature was lower than 243°C, and was in a supercritical state when it was higher than 243°C. The comparisons of reaction autogenous pressure and corresponding saturated vapor pressure under different reaction temperatures are shown in Figure 1A. With the increase in reaction temperatures, the autogenous pressure increases gradually, especially when the reaction temperatures are higher than 220°C, showing a sharp increase trend. Meanwhile, the reaction autogenous pressure was close to the saturated vapor pressure when the reaction temperatures were increased from 180°C to 220°C. There appeared to be an increasing gap between the reaction autogenous pressure and the corresponding saturated vapor pressure when the reaction temperatures were further improved (240°C and 260°C). These results indicated that fewer gas products were generated when the reaction temperatures were not higher than 220 °C, while further increasing the reaction temperature gradually generated a large number of gas products. To further investigate the composition of the gas products, they were collected when the reactor was cooled quickly by placing it in water after the reaction at 240°C and analyzed by GC equipped with a TDX-01 filler column. As shown in Figure 1B, CO_2_ was detected as the predominant gaseous product in the GC chromatogram, with small amounts of CO, CH_4,_ and H_2_. During biomass liquefaction, gases were generally found to be formed in the early stages of decomposition of the three main constituents of lignocellulosic biomass: cellulose, hemicelluloses, and lignin. The formation of CO_2_ and CO was caused by the decomposition of oxygen-containing groups in the biomass or in reaction intermediates (C=O, C-O-C, COOH, etc.) via decarbonylation and decarboxylation. However, the formation of CH_4_ was caused by the decomposition of methoxyl groups (-O-CH_3_) because the methanation of CO and CO_2_ is negligible at low temperatures (Yakaboylu et al., 2015; Li et al., 2019). Therefore, the gap between the autogenous pressure of the reaction and the corresponding saturated vapor pressure at high temperatures may be caused by additional tobacco pyrolysis and reaction intermediates.

**FIGURE 2:**
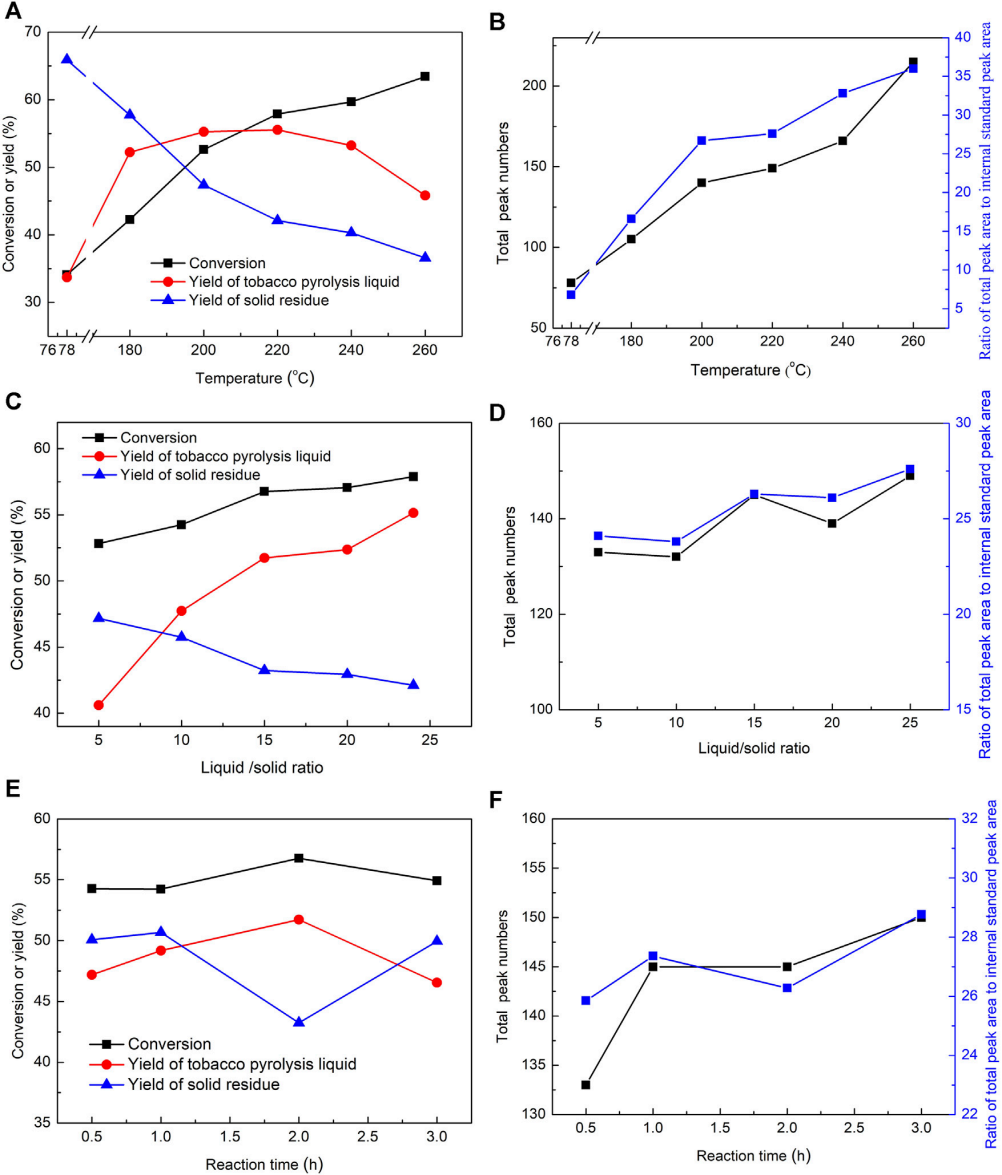
The influences of reaction temperature, liquid/solid mass ratio, and reaction time on the conversion, yield, and total peak numbers in the GC-MS chromatogram of tobacco pyrolysis liquid in the pyrolysis reaction of tobacco. Reaction conditions: **(A,B)** liquid/solid mass ratio = 25, 2 h, **(C,D)** 220°C, 2 h, **(E,F)** 220°C, liquid/solid mass ratio = 15.

The summarized reaction results, including the conversion, yield, and total peak numbers in the GC-MS chromatogram of the tobacco pyrolysis liquid, are shown in Figure 2. The tobacco conversion showed a gradual improvement trend with the increase in reaction temperatures, while the yields of solid residues were correspondingly decreased (Figure 2A). This indicated that higher reaction temperatures were conducive to the decomposition and transformation of tobacco. The yields of tobacco pyrolysis liquid displayed a slight increase when the reaction temperature was increased from 180°C to 220°C and an obvious decrease when this was further increased, reaching the maximum at 220°C. Considering that the pyrolysis reaction was a continuous reaction, it is inferred that further deep pyrolysis of tobacco pyrolysis liquid may occupy an increasing proportion at higher reaction temperatures, leading to a decrease in tobacco pyrolysis liquid yields and the production of small molecular gas products, which also coincided with the increasing gap between the autogenous pressure of the reaction and the corresponding saturated vapor pressure at high temperatures (Figure 1). At the same time, total peak numbers, and the ratio of the total peak area to the internal standard peak area in the GC-MS chromatogram of the tobacco pyrolysis liquid exhibited a gradual trend of improvement with the increase of the reaction temperature (Figure 2B), indicating that the number and contents of volatile components in the tobacco pyrolysis liquid were improved with the increase of the reaction temperature. Overall, the reaction temperature had a significant effect on the pyrolysis and transformation of tobacco, as it promoted the conversion of tobacco and the formation of volatile components; however, an excessive reaction temperature led to deep pyrolysis and decreased the yield of the tobacco pyrolysis liquid. The maximum yield was achieved at 220°C.

Second, the influence of the liquid/solid mass ratio was further examined under the conditions of 220°C and a 2-h reaction time, and the results are displayed in Figures 2C, D. The conversion and yields of the tobacco pyrolysis liquid showed a sharp rise when the liquid/solid mass ratio was increased from 5 to 15, and a slow increment when it was further increased to 25. Accordingly, the yields of solid residues showed an opposite trend. This indicated that increasing the amount of solvent was advantageous in advancing the pyrolysis reaction, possibly because a high liquid/solid mass ratio can generate sufficient free radicals to promote a more thorough pyrolysis reaction. However, continuously increasing the liquid/solid mass ratio gradually diminishes the effectiveness of the pyrolysis reaction. Herein, the optimal liquid/solid mass ratio is 15 from an economic point of view, although a higher liquid/solid mass ratio contributes to tobacco conversion and the yield of tobacco pyrolysis liquid. In addition, total peak numbers and the ratio of the total peak area to the internal standard peak area in the GC-MS chromatogram of the tobacco pyrolysis liquid presented a small fluctuation as the liquid/solid mass ratio increased, suggesting that the liquid/solid mass ratio had less effect on the depth of the pyrolysis reaction. Therefore, a high liquid/solid mass ratio could promote the formation of tobacco pyrolysis liquid, but the depth of the pyrolysis reaction was almost unchanged.

Finally, Figures 2E, F show the influence of reaction time on the pyrolysis reaction. The conversion and the yields of tobacco pyrolysis liquid first increased and then decreased, reaching a peak at 2 h, while the yields of solid residues showed an opposite trend. Considering that the macromolecule pyrolysis reaction and the small-molecule polymerization reaction are reciprocal reactions, it is inferred from the decrease in the yield of the tobacco pyrolysis liquid that the polymerization reaction may occupy an increasing fraction of the pyrolysis reaction as the reaction time increases. The total number of peaks and the ratio of the total peak area to the internal standard peak area in the GC-MS chromatogram of the tobacco pyrolysis liquid showed a slight wave when the reaction time was longer than 0.5 h.

In summary, the reaction temperature had a significant impact on the pyrolysis reaction of tobacco. Higher reaction temperatures were conducive to the pyrolysis conversion of tobacco, resulting in a high tobacco conversion and a high amount and content of volatile components in the tobacco pyrolysis liquid. However, the yield of tobacco pyrolysis liquid also decreased further at too-high reaction temperatures (higher than 220 ^o^C). The liquid/solid mass ratio was beneficial for the yield of tobacco pyrolysis liquid but had little effect on the depth of the pyrolysis reaction. The optimal reaction conditions for the preparation of tobacco pyrolysis liquid were found to be a temperature of 220°C, a liquid/solid mass ratio = 15, and a 2-h reaction time.

### 3.2 Volatile component analysis of tobacco pyrolysis liquid prepared at different reaction temperatures

**FIGURE 3:**
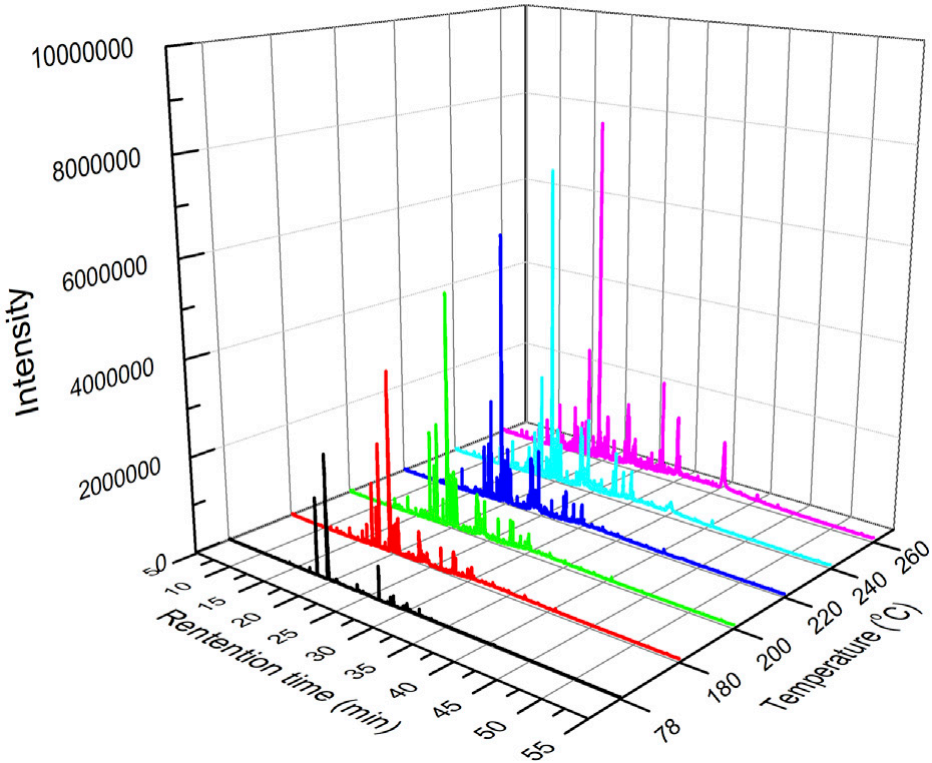
GC-MS chromatogram of tobacco pyrolysis liquids prepared at different temperatures.

The analysis of pyrolysis products under different reaction temperatures was examined, considering that the reaction temperature is essential for the pyrolysis reaction of tobacco. The GC-MS chromatogram of tobacco pyrolysis liquids prepared at different temperatures is shown in Figure 3. This demonstrated an obvious improvement in peak height and number with increasing reaction temperature, suggesting that the amount and total concentration of pyrolysis volatile products also increased.

The GC-MS chromatographic peaks were further qualitatively determined with a matching degree greater than 80% as a standard, and the peak area ratio of compounds to the internal standard was used as a semi-quantitative analysis. The results are shown in Table 1. The qualitative results showed that the pyrolysis products were mainly esters, nitrogen-containing heterocycles, phenols, alcohols, aldehydes, ketones, and other substances; these types of compounds are common flavoring substances. Therefore, the tobacco pyrolysis liquid showed potential application in the aroma enhancement of heated cigarettes. Moreover, the peak area ratios of the compound to the internal standard in the tobacco pyrolysis liquid prepared at different temperatures were also significantly different, indicating that the reaction temperatures could significantly change the distribution of products and the depth of the pyrolysis reaction.

**TABLE 1:** Semi-quantitative results of tobacco pyrolysis liquid prepared at different temperatures.

Type of compounds	Reaction time (min)	Chemical compounds	Peak area ratio of the compound to the internal standard					
			260°C	240°C	220°C	200°C	180°C	78°C
Ester	10.433	Butanoic acid, 2-hydroxy-, ethyl ester	11.93%	5.74%	2.08%	1.75%	-	-
	11.799	Tetraethyl silicate	0.22%	0.10%	0.06%	0.07%	-	-
	13.536	2-Furancarboxylic acid, ethyl ester	10.80%	0.42%	0.09%	-	-	-
	13.654	Levulinate ethyl ester	40.56%	30.68%	19.66%	0.85%	-	-
	15.763	2-Butenedioic acid (Z)-, diethyl ester	0.22%	0.29%	-	-	-	-
	15.947	Butanedioic acid, diethyl ester	40.42%	16.18%	-	-	-	-
	16.117	2-Butenedioic acid (E)-, diethyl ester	23.89%	31.50%	5.03%	0.10%	-	-
	16.438	Glutaric acid, ethyl 2-ethylbutyl ester	7.42%	0.17%	-	-	-	-
	17.58	Butanedioic acid, hydroxy-, diethyl ester	1.65%	3.31%	2.84%	-	-	-
	17.964	Benzoic acid, 2-hydroxy-, ethyl ester, internal standard	100.00%	100.00%	100.00%	100.00%	100.00%	100.00%
	18.289	5-Oxotetrahydrofuran-2-carboxylic acid, ethyl ester	7.07%	8.64%	6.06%	5.47%	-	-
	20.727	2-Pyrrolidinecarboxylic acid-5-oxo-, ethyl ester	93.28%	79.47%	50.97%	-	0.16%	-
	21.545	Ethyl 3-hydroxybenzoate	13.49%	-	-	-	-	-
	23.59	Ethyl.α.-D-glucopyranoside	5.75%	207.71%	3.71%	5.16%	0.21%	
	24.051	1,4-Benzenedicarboxylic acid, diethyl ester	0.35%	-	1.12%	-	-	-
	25.853	Tetradecanoic acid, ethyl ester	0.31%	-	-	-	-	-
	26.413	p-Hydroxycinnamic acid, ethyl ester	0.26%	0.23%	0.14%	-	-	-
	27.678	Ethyl (2E)-3-(4-hydroxy-3-methoxyphenyl)-2-propenoate	0.33%	0.25%	-	-	-	-
	28.099	Dibutyl phthalate	20.18%	27.99%	22.00%	24.54%	19.09%	-
	28.419	Hexadecanoic acid, ethyl ester	75.74%	2.61%	1.43%	1.28%	0.81%	-
	29.614	Heptadecanoic acid, ethyl ester	0.21%	0.15%	-	-	-	-
	30.439	9,12-Octadecadienoic acid, ethyl ester	-	1.24%	0.68%	0.63%	-	-
	30.516	Ethyl 9,12,15-octadecatrienoate	2.75%	1.71%	0.98%	0.85%	0.57%	-
	30.766	Octadecanoic acid, ethyl ester	0.92%	0.49%	4.79%	0.22%	0.17%	-
Nitrogen-containing heterocycles	7.764	2-Methylpyridine	-	-	0.07%	-	-	-
	13.802	Ethanone, 1-(1H-pyrrol-2-yl)-	0.12%	0.13%	0.09%	0.10%	-	-
	14.386	3-Pyridinol	13.26%	14.50%	0.16%	-	-	-
	15.478	2-(1H)-Pyridinone, 3-methyl-	0.21%	2.66%	-	-	-	-
	18.441	Indole	0.10%	0.11%	-	-	-	-
	19.46	Nicotine	532.76%	502.20%	484.61%	453.23%	422.69%	357.27%
	22.577	2,3′-Dipyridyl	4.27%	2.27%	2.69%	0.18%	0.17%	-
	28.8	9H-Pyrido [3,4-b]indole	0.15%	0.17%	0.09%	-	0.11%	-
Phenol	11.915	Phenol	4.31%	-	-	-	-	-
	13.948	p-Cresol	0.19%	-	-	0.32%	-	1.96%
	16.289	Catechol	1.27%	-	0.29%	-	-	-
	19.696	4-Ethylcatechol	23.25%	-	-	-	-	-
Alcohol	9.334	2-Furanmethanol	0.09%	4.54%	2.13%	0.15%	-	-
	13.276	Benzylalcohol	0.10%	0.09%	-	-	-	-
	14.934	Phenylethyl Alcohol	0.32%	-	-	-	-	-
	26.699	Rishitin	0.29%	0.27%	0.19%	-	-	-
Aldehyde	11.702	5-Methyl-2-furancarboxaldehyde	25.88%	19.46%	10.15%	9.53%	0.19%	-
	12.695	1H-Pyrrole-2-carboxaldehyde	0.09%	0.17%	0.10%	-	-	-
	16.92	5-Hydroxymethylfurfural	0.52%	2.62%	2.60%	4.81%	3.17%	5.69%
Ketone	13.021	1,2-Cyclopentanedione, 3-methyl-	6.60%	0.15%	0.07%	-	-	-
	23.818	Megastigmine	-	-	-	-	-	1.78%
Acid	27.978	n-Hexadecanoic acid	0.21%	0.45%	0.40%	-	0.27%	8.71%
	30.09	9,12-Octadecadienoic acid (Z,Z)-	-	0.11%	0.08%	0.09%	-	2.59%
	30.166	9,12,15-Octadecatrienoic acid, (Z,Z,Z)-	-	0.22%	0.20%	0.22%	0.12%	8.14%

Among the ester compounds, there were a large number of ethyl ester compounds, such as 2-hydroxybutanoic acid ethyl ester, 2-furancarboxylic acid ethyl ester, levulinate ethyl ester, butanedioic acid diethyl ester, dibutyl phthalate, hexadecanoic acid ethyl ester, and 5-oxo-2-pyrrolidinecarboxylic acid ethyl ester. According to the literature (Shimada et al., 2008; Lu et al., 2009; Long et al., 2017), 2-hydroxybutanoic acid, 2-furancarboxylic acid, and butanedioic acid could be generated from the pyrolysis reaction of sugars such as cellulose existed in tobacco leaves. Therefore, the ethyl ester compounds in the tobacco pyrolysis liquid may mainly originate from the esterification reaction of cellulose and other sugar pyrolysis products and tobacco inclusion compounds with ethanol. Moreover, the contents of 2-hydroxybutanoic acid ethyl ester, levulinate ethyl ester, and butanedioic acid diethyl ester in the tobacco pyrolysis liquid showed an improving trend with increasing reaction temperature, indicating that high reaction temperatures were conducive to the pyrolysis reaction and the subsequent esterification reaction.

**FIGURE 4:**
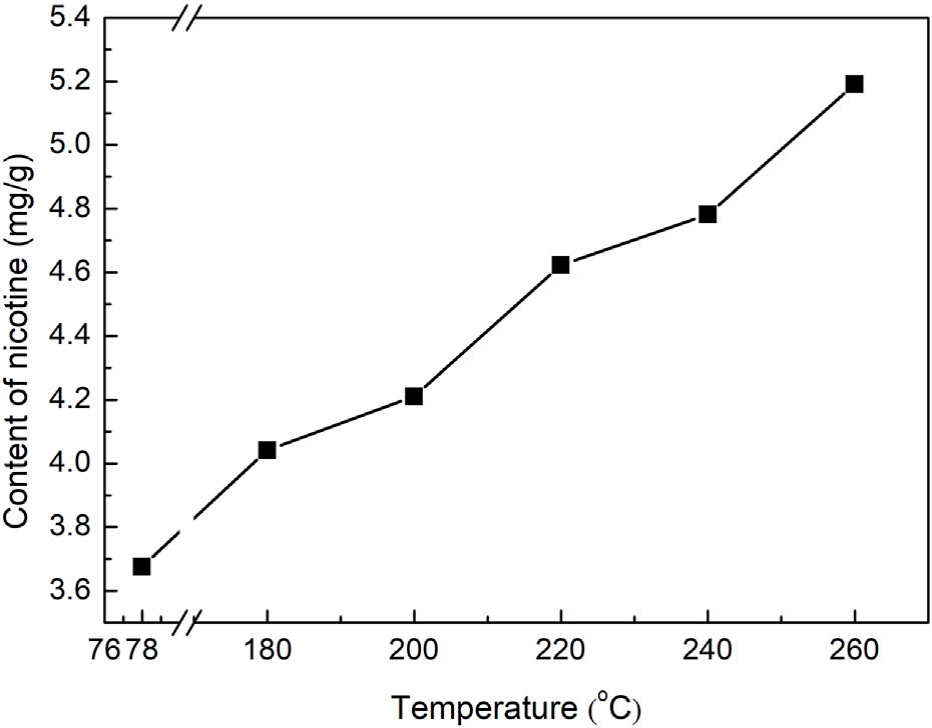
The content of nicotine in tobacco pyrolysis liquid prepared at different temperatures.

Nicotine was the main nitrogen-containing heterocycle in the tobacco pyrolysis liquid, which was also the highest in tobacco pyrolysis liquids prepared under different temperatures. The peak area ratios of nicotine to the internal standard improved with increasing reaction temperatures. As the unique alkaloid in tobacco, nicotine content was an extremely important index of tobacco pyrolysis liquid. In our study, the accurate content of nicotine in the tobacco pyrolysis liquid was further quantitatively analyzed by GC methods, and the results are displayed in Figure 4. The nicotine content in the tobacco pyrolysis liquid was higher than that of the tobacco extract prepared at 78°C and showed a remarkable rising trend with the increase of the reaction temperature, which was also consistent with the change of the peak area ratios (Table 1). Nicotine in tobacco is distributed in a dense structure composed of cellulose, hemicellulose, and lignin and exists mainly as a salt by combining inorganic or organic acids, with a small amount in the free state. Higher reaction temperatures could promote the pyrolysis reaction of cellulose in subcritical/supercritical ethanol, decomposing the dense structure of tobacco leaves and leading to the release of nicotine. On the other hand, higher reaction temperatures may also benefit the decomposition of nicotine salts in tobacco. Both of these two situations would to the higher nicotine contents in tobacco pyrolysis liquids under high reaction temperatures. Considering that heated cigarettes require the addition of nicotine to enhance satisfaction, tobacco pyrolysis liquids with a high nicotine content were more suitable for heated cigarettes than tobacco extracts. Moreover, among other nitrogen-containing compounds, 2,3′-dipyridyl, another tobacco alkaloid, also showed similar trends to nicotine.

Phenolic compounds, including 4-ethylcatechol and phenol, were mainly presented at 260°C. Lignin degradation produces phenolic compounds (Singh et al., 2021; Li et al., 2022), implying that higher reaction temperatures may be favorable for the depolymerization of lignin. In terms of alcohol, aldehyde, and ketone compounds, 2-furan methanol, 5-hydroxymethylfurfural, 5-methyl-2-furancarboxaldehyde, and 3-methyl-1,2-cyclopentanedione were the main products in tobacco pyrolysis liquids. According to the literature (Baker and Bishop, 2004; Baker and Bishop, 2005; Baker et al., 2005; Mitsui et al., 2015; Li et al., 2021), these compounds were the pyrolysis products of sugars such as cellulose, indicating that sugar substances may undergo pyrolysis reactions in the supercritical system and generate small molecule substances. On the other hand, these compounds were important aroma substances in smoke, which can enhance the aroma content and improve the taste, further indicating the potential fragrance ability of tobacco pyrolysis liquid.

### 3.3 Analysis of solid residues reacted at different reaction temperatures

**FIGURE 5:**
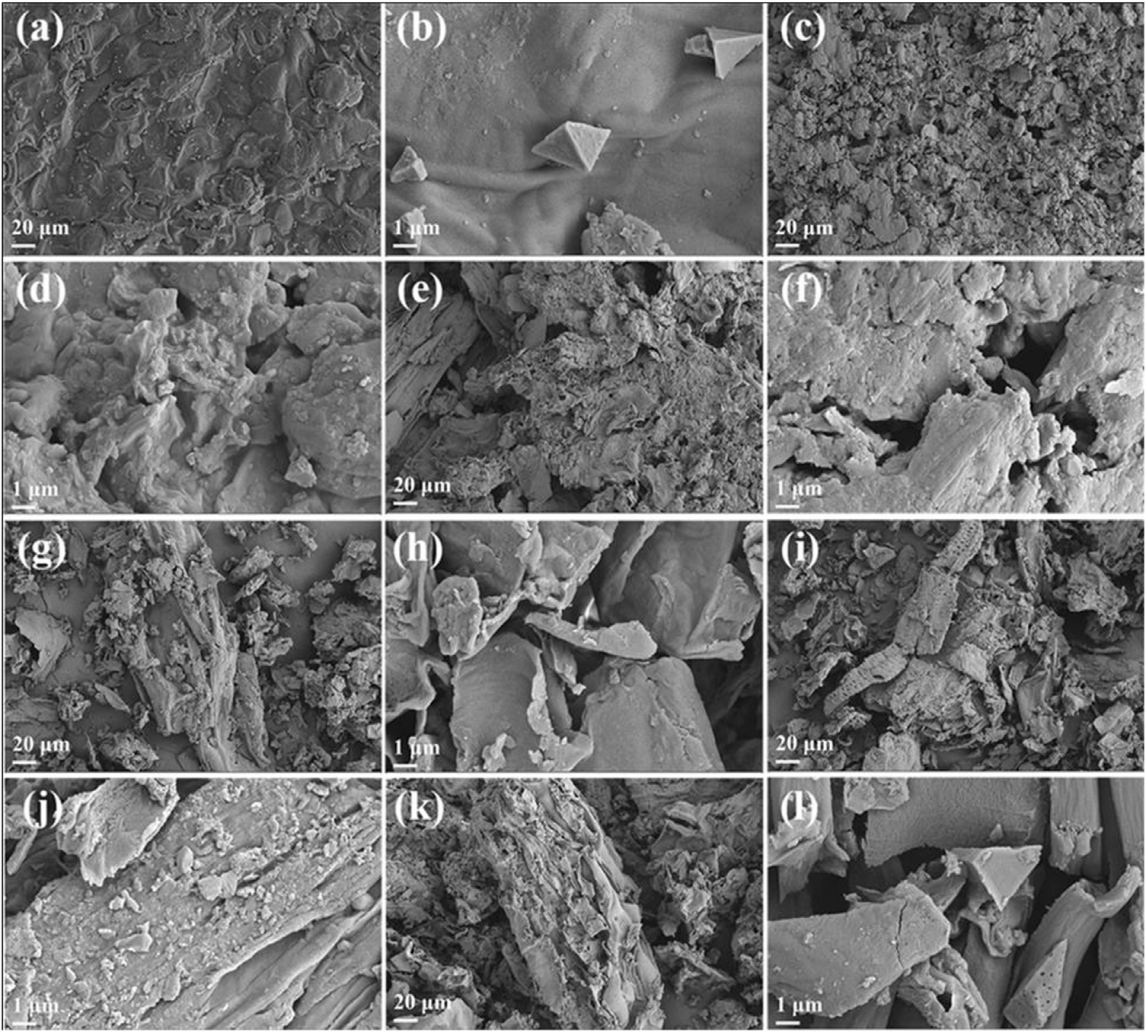
SEM images of solid residues that reacted at different temperatures. **(A,B)** 78°C, **(C,D)** 180°C; **(E,F)** 200°C; **(G,H)** 220°C; **(I,J)** 240°C; **(K,L)** 260°C.

To gain a deeper understanding of the pyrolysis reaction of tobacco, the solid residues obtained at different reaction temperatures were further characterized by SEM, elemental analysis, and FT-IR. The SEM images in Figure 5 depict the micromorphology of the solid residues. As a control group, Figures 5A, B showed the micromorphology of the solid residues after reaction at atmospheric pressure treatment and 78°C. It is evident that the surface of tobacco residues after atmospheric pressure treatment has a dense structure composed of particles. Further magnification images confirmed that the surface morphology of the samples is smooth and dense, indicating that atmospheric pressure treatment could not damage the dense structure on the surface of tobacco leaves. In contrast, the micromorphology of solid residues after reaction in subcritical/supercritical ethanol under different temperatures, as displayed in Figures 5C–L, showed significant changes compared to the control group. The externally dense microstructure appeared damaged, becoming rough and exhibiting pyrolysis, holes, and fragments. All indicated that sub/supercritical ethanol treatment could significantly disrupt the surface structure of tobacco leaves. The severe destruction of the surface structure was conducive to the subsequent pyrolysis and liquefaction of the tobacco. Furthermore, as the reaction temperature increased, the degree of surface structure destruction increased, which accelerated the pyrolysis and liquefaction of tobacco leaves.

The elemental analysis results of the tobacco residues are listed in Table 2. With the increase in the reaction temperature, the oxygen and hydrogen contents in the tobacco residues gradually decreased, while the carbon and nitrogen contents first decreased and then increased, reaching the minimum value at 200°C. When the reaction temperature was increased to 200°C, the contents of carbon, hydrogen, oxygen, and nitrogen in the tobacco residues gradually decreased, indicating that the inclusions in the tobacco were gradually dissolved and reacted with the increase of the reaction temperature, which was also consistent with the gradual increase of the yield of the tobacco pyrolysis liquid. When the reaction temperature was further increased to 260°C, the carbon content had a significant increase, indicating that the carbonization degree of the tobacco residue gradually increased at high temperatures.

**TABLE 2:** Elemental analysis results of tobacco residues.

Reaction temperatures (^o^C)	Element contents (%)			
	C	H	O	N
78	36.87	5.00	39.48	2.34
180	35.04	4.81	39.46	1.81
200	32.42	4.41	38.36	1.36
220	32.97	4.28	37.72	1.57
240	32.90	4.18	36.54	1.57
260	35.60	4.16	34.63	1.76

**FIGURE 6:**
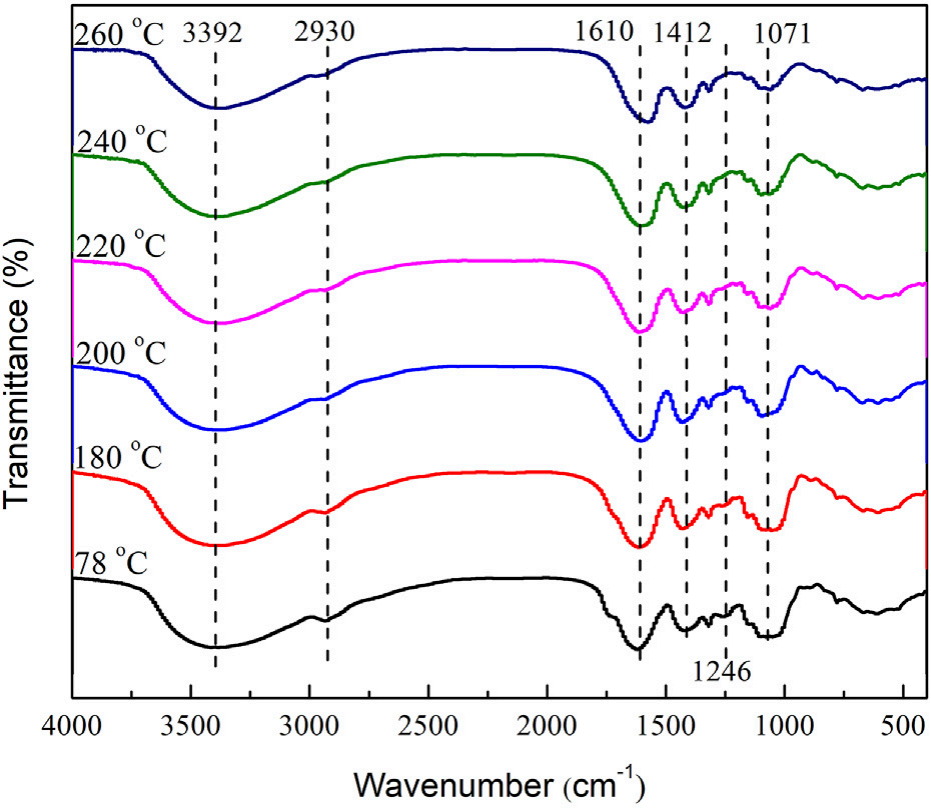
FT-IR spectra of solid residues after reaction at different temperatures.

The FT-IR spectra of tobacco residues obtained at different reaction temperatures are shown in Figure 6. According to the literature (Kim et al., 2012), the absorption peak at 3392 cm^-1^ was attributed to the stretching vibration of O-H in hydroxyl compounds (such as sugars, phenols, alcohols, and water). As the reaction temperatures increased, the O-H stretching vibration peak in the solid residue gradually weakened, mainly due to dehydration and hydroxyl bond cleavage in tobacco leaves with increasing temperature. However, the O-H stretching vibration peak in the residue remained relatively strong, possibly due to the generation of H^.^, CH3^.^, and OH^.^ radicals from ethanol under sub/supercritical conditions, which reacted with the products to form hydroxyl-containing substances (Tao et al., 2014). The absorption peaks at 2,930 cm^−1^ and 1430 cm^−1^ were attributed to the stretching or deformation vibrations of methyl and methylene C-H (Chen et al., 2016). The absorption peak at 1610 cm^-1^ was attributed to the stretching vibration of C=C in aromatic compounds in tobacco leaves (Yan et al., 2018). When tobacco leaves were treated under sub/supercritical conditions, a series of aromatic compounds (heterocyclic) were generated by the pyrolysis reaction. When the temperature was in the subcritical range, the peak intensity of the C=C stretching vibration of the aromatic compounds was relatively stable. When the temperature was raised to 220°C, the peak intensity of the C=C stretching vibration of aromatic compounds weakened, which was consistent with the increase in the content of aromatic hydrocarbons in the liquid products at 220°C. The characteristic absorption peak of cellulose in tobacco leaves was at 1062–1031 cm^−1^ (Tao et al., 2014). During the sub/supercritical ethanol liquefaction of tobacco leaves, there was a strong C-O-C stretching vibration peak (1071 cm^−1^) in the residue when the temperature was low. As the processing temperature increased, the C-O-C peak gradually weakened, and at the same time, the C-H stretching vibration absorption peak (2,930 cm^−1^) and the C-OH absorption peak (1246 cm^−1^) also gradually disappeared, possibly due to the thermal dehydration and cleavage of the C-O-C glycosidic bond in cellulose in tobacco leaves. In summary, as the reaction temperatures increased, the characteristic infrared peak intensities of organic structures such as methyl, aromatic ring skeleton, and cellulose in tobacco leaves gradually weakened, indicating an increase in the degree of depolymerization of tobacco leaves with increasing temperatures.

### 3.4 Application of tobacco pyrolysis liquid to the aroma enhancement of heated cigarettes

The tobacco pyrolysis liquid prepared under the selected optimal process conditions (220°C, liquid/solid mass ratio of 15, and reaction time of 2 h) was evaluated for its aroma-enhancing effect in heated cigarettes. GC-MS/MS was used to analyze the changes in the content of major aroma components in the smoke of heated cigarettes before and after the addition of tobacco pyrolysis liquid. At the same time, blank cigarettes and injection tobacco extracts (prepared at 78°C) were selected as control samples. The main aroma components and their levels in the smoke are shown in Table 3. The total particulate matter (TPM) content of the blank cigarette was 20 mg/cig, which increased to 20.6 mg/cig after heating the tobacco extract. However, after the addition of the tobacco pyrolysis liquid, the TPM content significantly increased to 22.4 mg/cig. This indicates that the large amounts of volatile substances contained in the tobacco pyrolysis liquid can be transferred to the smoke of heated cigarettes under actual use conditions, significantly increasing the release of TPM. Moreover, the nicotine content increased slightly to 0.415 mg/cig, which was also associated with the high nicotine content of the tobacco pyrolysis liquid.

**TABLE 3:** The content of major aroma compounds in the smoke of heated cigarettes before and after the addition of tobacco pyrolysis liquid.

Content	Blank cigarettes	Cigarettes infused with tobacco extract prepared at 78°C	Cigarettes infused with tobacco pyrolysis liquid prepared at 220°C
TPM (mg/cig)	20	20.6	22.4
Nicotine (mg/cig)	0.410	0.410	0.415
Aroma compounds (ng/cig)			
Acid	155121.10	140419.59	168083.98
Ketone	128208.73	124712.27	133531.13
Alcohol	68055.86	55440.40	68808.90
olefin	19469.38	18076.27	22737.97
Aldehyde	10877.10	10168.62	13070.58
Phenol	7370.38	5617.13	7446.60
Ester	4781.75	3861.21	5675.10
Nitrogen-containing compounds	1127.13	1180.97	1365.24
oxygen-containing compounds	261.12	273.84	300.96
Total	395272.55	359750.29	421020.46

In the method of aroma compound analysis, mass spectrometry was used in the MRM scanning mode, and 157 common aroma substances belonging to 9 categories were selected, the contents of which are shown in Table 2. These categories include acids, ketones, alcohols, olefins, aldehydes, phenols, esters, nitrogen-containing compounds, and oxygen-containing compounds. Compared to the blank sample, the content of nitrogen-containing compounds and oxygen-containing compounds in the smoke slightly increased after the addition of tobacco extract, while the content of acids, ketones, alcohols, olefins, aldehydes, phenols, esters, and the total content of aroma compounds all decreased to some extent. This indicates that the tobacco extract prepared by atmospheric pressure treatment cannot effectively achieve the purpose of aroma enhancement in heated cigarettes. After the addition of tobacco pyrolysis liquid, the contents of all nine categories of aroma compounds in the smoke increased to varying degrees, and the total content of aroma substances increased. This indicated that tobacco pyrolysis liquid could significantly increase the release of aroma substances in the smoke of heated cigarettes and have a significant aroma-enhancing effect.

## 4 Conclusion

Tobacco pyrolysis liquids containing large amounts of volatile components could be prepared in subcritical/supercritical ethanol. Reaction temperatures had the most significant impact on the tobacco pyrolysis reaction, and higher reaction temperatures promoted the pyrolysis conversion of tobacco, resulting in high tobacco conversion and high amounts and contents of volatile components in tobacco pyrolysis liquids. However, the yield of tobacco pyrolysis liquid decreased after the reaction temperatures exceeded 220°C. The liquid/solid mass ratio was beneficial for the yield of tobacco pyrolysis liquid but had little effect on the depth of the pyrolysis reaction. The optimal reaction conditions for the preparation of tobacco pyrolysis liquid were found to be a temperature of 220°C, a liquid/solid mass ratio = 15, and a reaction time of 2 h. The content of ester compounds significantly increased in the tobacco pyrolysis liquid, and the content of nicotine also increased significantly with the increase in reaction temperature. Sub/supercritical ethanol treatment significantly disrupted the surface structure of tobacco, indicating an increase in the degree of tobacco depolymerization with increasing temperature. The analysis of aroma compounds in the smoke of heated cigarettes indicated that the tobacco pyrolysis liquid could significantly increase the release of aroma substances and have a significant aroma-enhancing effect.

## Data Availability

The original contributions presented in the study are included in the article/supplementary material, further inquiries can be directed to the corresponding authors.
